# Assessment of genotoxicity and antigenotoxicity of an aqueous extract of *Cleistocalyx nervosum* var. *paniala* in *in vitro* and *in vivo* models

**DOI:** 10.2478/v10102-012-0033-2

**Published:** 2012-12

**Authors:** Suphachai Charoensin, Sirinya Taya, Sugunya Wongpornchai, Rawiwan Wongpoomchai

**Affiliations:** 1 Department of Biochemistry, Faculty of Medicine, Chiang Mai University, Chiang Mai, Thailand; 2 Department of Chemistry, Faculty of Science, Chiang Mai University, Chiang Mai, Thailand; 3 Department of Biochemistry, School of Medical Science, University of Phayao, Phayao, Thailand; * These authors contributed equally to this work

**Keywords:** *Cleistocalyx nervosum* var. *paniala*, acute toxicity, ames test, liver micronucleus assay

## Abstract

*Cleistocalyx nervosum* var. *paniala*, an edible fruit found in Northern Thailand, contains high amounts of phenolic compounds with *in vitro* antioxidant activity. The aqueous extract of the ripe fruit was evaluated for its safety and beneficial effects using genotoxicity and toxicity tests. The *C. nervosum* extract was not only non-mutagenic in *Salmonella typhimurium* strains TA98 and TA100 in the presence and absence of metabolic activation, but exhibited also moderate antimutagenic effects against aflatoxin B1 and 2-amino-3,4-dimethylimidazo[4,5-*f*]quinoline-induced mutagenesis. Electrospray ionization-mass spectrometric analysis revealed the major anthocyanins, which included cyanidin-3,5-diglucoside, cyanidin-3-glucoside and cyanidin-5-glucoside. The administration of *C. nervosum* at concentration of 5,000 mg/kg bw did not induce acute toxicity in rats. A liver micronucleus test was performed to detect clastogenicity and anticlastogenicity. The extract in the dose of 1,000 mg/kg did not cause micronucleus formation in the liver of rats. Furthermore, in rats administered 100–1,000 mg/kg of the extract, no anticlastogenic effect against diethylnitrosamine-induced hepatic micronucleus formation was observed. These studies provide data concerning the safety and antimutagenic potency of an aqueous extract of *C. nervosum* fruit.

## Introduction

Anthocyanins occur ubiquitously in flowering plants and confer the bright red, blue and purple colors to fruits and vegetables. Epidemiological studies have suggested that the consumption of anthocyanins lowers the risk of cardiovascular disease, diabetes, arthritis and cancer, due at least in part to anti-oxidant and anti-inflammatory activities (Prior & Wu, 2006). Potential cancer chemopreventive activities of anthocyanins revealed in *in vitro* studies included radical scavenging activity, stimulation of phase II detoxifying enzymes, reduced cell proliferation, inflammation, angiogenesis, invasiveness and induction of apoptosis and differentiation (Caillet *et al.*, 2012; Galvano *et al.*, 2004). Anthocyanins were shown to exhibit anticarcinogenic activity against multiple cancer cell types *in vitro* and tumor types *in vivo* (Galvano *et al.*, 2004; Stoner *et al.*, 2008).

*Cleistocalyx nervosum* var. *paniala* family Myrtaceae is a native plant found in Northern Thailand, with an orange-red fruit that is commonly consumed either as fresh fruit or is used in fruit products. Previous studies have reported that cyanidin-3-glucosides were found in the ripe fruit of *C. nervosum* (Jansom *et al.*, 2008). There are but few data concerning the biological activity of *C. nervosum* fruit *in vitro* or *in vivo*, though *Cleistocalyx operculatus*, which belongs to the same genus, has been investigated for its biological activities. The buds of *C. operculatus* are used in various beverages in Southern China (Ye *et al.*, 2004a). Dung *et al.* studied the chemical composition, antimicrobial and antioxidant activities of the essential oil and ethanol extract of *C. operculatus* buds. 2’,4’-Dihydroxy-6’-methoxy-3’,5’-dimethylchalcone (DMC), isolated from buds of *C. operculatus,* was found to inhibit significantly the growth of human liver cancer SMMC-7721 cells (Ye *et al.*, 2004b; Dung *et al.*, 2008).

In studies of natural products, it is quite common to characterize their benefits and hazards to humans through extrapolation of effects from *in vitro* and animal studies. The goal of mutagenicity testing is to identify genotoxic or carcinogenic effects of the test compound (Rao *et al.*, 2004). The standard short-term genotoxicity tests include bacterial mutation assays (Ames test), micronucleus tests and chromosomal aberration tests. These tests can be performed rapidly and are relatively inexpensive. They are appropriate for estimating the carcinogenic effects of the chemicals and as guidelines for long-term carcinogenicity tests (Kirkland *et al.*, 2006). Such genotoxicity tests have also been modified for detecting antigenotoxicity of natural products (Inboot *et al.*, 2012).

This study aimed at the evaluation of *in vitro* mutagenicity and antimutagenicity of *C. nervosum* var. *paniala* extracts by means of the Ames test. Clastogenic and anticlastogenic effects were also observed in rat liver using the micronucleus assay. Because *C. nervosum* is extensively consumed as a fresh fruit, it is desirable to determine the limits of toxicity for large amounts. Acute toxicity test was therefore also assessed in rats.

## Materials and methods

### Chemicals

All chemicals and solvents used were of analytical grade. The standard mutagens used included 2-(2-furyl)-3-(5-nitro-2-furyl)-acrylamide (AF-2), 2-aminoanthracene (2-AA), and 2-amino-3,4-dimethylimidazo[4,5-*f*] quinoline (MeIQ); these were purchased from Wako Pure Chemicals Industries, Ltd. (Osaka, Japan). Aflatoxin B1 (AFB1) was purchased from Sigma Chemical Co. (St. Louis, MO, USA). Cyanidin-3-*O*-glucoside was purchased from Apin Chemicals Ltd. (Oxfordshire, UK). Collagenase type IV and 4’,6-diamidino-2-phenylindole (DAPI) were purchased from Invitrogen Corporation (USA). All solvents used in the analysis of anthocyanin constituents were of HPLC grade.

### Plant material

Ripe fruits of *Cleistocalyx nervosum* var. *paniala* were collected during July-August, 2008 from Tambon Choeng Doi, Amphur Doi Saket, Chiang Mai, Thailand. This plant was identified and confirmed by comparing it with voucher specimens of known identities (QBG 7290, QBG 17340, QBG 25139) deposited at the Queen Sirikit Botanic Garden, Chiang Mai, Thailand. The pulp was manually separated from seeds, weighed and stored at –20 °C until use.

### Sample preparation

One hundred gram of *C. nervosum* pulp was ground with 50 ml of distilled water using a blender. The mixture was centrifuged at 1,000 g for 15 min and filtered through a filter paper. The filtrate was dried using a lyophilizer. The aqueous extract was a purplish-red solid with final yield equivalent to 8.48 g.

### Determination of total phenolic and flavonoid contents and condensed tannins

The total phenolic content was determined via the Folin-Ciocalteu reagent method (Singleton *et al.*, 1999). The absorbance of the sample was measured at 765 nm, and results were expressed as mg gallic acid equivalent (GAE)/g fresh weight (fw). Total flavonoid content was determined using the aluminum chloride colorimetric method. The absorbance at 532 nm was used and expressed as mg catechin equivalent (CE)/100 g fw (Maksimovic *et al.*, 2005). Finally, the condensed tannin was assessed by the vanillin assay (Butler *et al.*, 1982). After spectrophotometric analysis at 550 nm, the amount of condensed tannins was calculated as mg CE/100 g fw.

### Quantification of anthocyanins by High-Performance Liquid Chromatography (HPLC) and Liquid Chromatography-Electrospray Ionization-Mass Spectrometry (LC-ESI-MS)

The *C. nervosum* aqueous extract was dissolved in 0.1% formic acid in methanol and filtered through filter paper and the remaining methanol was evaporated under reduced pressure.

A 2.1 × 150 mm Halo column (Agilent Technologies, USA) with a particle size of 2.7 µm was used. The mobile phase consisted of water containing 0.5% formic acid (solvent A) and methanol (solvent B), with gradient elution started at 85:15 (A:B). The HPLC effluent was passed through the photodiode array detector (PAD), which was set to monitor at wavelengths of 254 and 520 nm. After separation, the HPLC effluent was delivered into a single quadrupole mass spectrometer (Agilent Technologies, USA) via orthogonal atmospheric pressure ionization (API)**-**electrospray interface. The optimum electrospray ionization (ESI) conditions were as follows: ionization mode, positive; nebulizer pressure, 32 psi; drying gas flow rate, 10 L/min; drying gas temperature, 350 °C; and capillary voltage, 4,000 V. Helium was used as a collision gas and a fragmentor voltage of 130 V was used for the collision-induced dissociation **(**CID**)**. The quadrupole temperature was 100 °C and the electron multiplier voltage was 2,650 V.

The relative contents of anthocyanins in the extract of *C. nervosum* were obtained by the LC-ESI-MS technique. Naphtholphthalein was used as an internal standard. Peak area normalization was utilized to determine the relative contents of the individual anthocyanin components. Three replicates of sample extract were analyzed.

### *In vitro* mutagenic and antimutagenic assays by Ames test

Both TA98 and TA100 of *Salmonella typhimurium* were kindly provided by Dr. Takehiko Nohmi, National Institute of Environmental Health Science, Japan. In brief, 20 µl of inoculum from the permanent culture were incubated in 10 ml of nutrient broth at 37 °C for 14 hr, until a bacterial concentration of approximately 1.2 × 10^9^ bacterial/ml was obtained.

According to the preincubation method described, the Salmonella mutation assay was performed under both non-metabolic and metabolic activation conditions (Maron and Ames, 1983). Briefly, solvent (negative control), 0.8–200 mg/ml of the extract, or standard mutagens as positive control were added to phosphate buffer or S9 mix. The mixtures were then preincubated with overnight culture of TA98 or TA100 strains at 37 °C for 20 min before adding a top agar containing 0.5 mM *L*-histidine/*D*-biotin. In all plates, the His^+^ revertant colonies were analyzed after 37 °C incubation for 48 hr. Triplicate plates per run were assayed.

As mentioned for the mutagenic assay, the preincubation technique was modified in the antimutagenic assay. In brief, the mixtures consisting of solvent alone or 100–200 mg/ml of extract in the presence of AFB1, MeIQ, or AF-2 were preincubated with overnight culture of TA98 strain with or without S9 mix at 37 °C for 20 min before top agar addition. The His^+^ revertant colonies were analyzed after 37 °C incubation for 48 hr. Triplicate plates per run were assayed. The number of counted revertant colonies was subtracted by the number of spontaneous revertants before calculating the percentage inhibition.

### Acute toxicity test

Male and female Wistar rats (150–180 g of weight) were obtained from the National Laboratory Animal Center, Thailand. They were housed under standard environmental conditions at a temperature of 24 ^o^C under 12 hr dark-light cycle, and allowed free access to drinking water and pelleted diet. The experimental protocol was approved by The Animal Ethics Committee of the Faculty of Medicine, Chiang Mai University.

The acute toxicity of the aqueous extract of *C. nervosum* was evaluated in rats using the fixed dose procedure (OECD, 2002). Rats were randomly divided into two groups of five animals per each sex. The aqueous extract at a single dose of 5,000 mg/kg and vehicle was given orally to the treated and control groups. On day 15, all rats were anesthetized with diethyl ether. The internal organs were excised and weighed. Gross pathological observations of the tissues were performed.

### *In vivo* clastogenic and anticlastogenic assays by rat liver micronucleus test

Male rats were orally administered 1,000 mg/kg of *C. nervosum* extract, while the control group was given 5% tween-80 as vehicle control for 21 days. All rats were subjected to partial hepatectomy on day 22. On day 26, the animals were sacrificed by two-step collagenase perfusion. In brief, anesthetized rats were inserted a canula into the portal vein to infuse the preperfusion medium to remove blood. Second, 0.05% collagenase medium, pH 7.4 was continuously perfused to isolate hepatocytes. Third, the liver was incised and then washed with phosphate buffer saline. Finally, the isolated hepatocytes were washed in 10% buffered formalin and re-suspended in the same solution. Hepatocyte suspensions were mixed with 4′,6-diamidino-2-phenylindole dihydrochloride (DAPI) stain solution for fluorescent microscopy. The number of micronucleated hepatocytes was counted and recorded, based on analysis of 2,000 hepatocytes from each animal under a fluorescent microscope. The criteria for micronucleated hepatocyte scoring were described elsewhere (Cliet *et al.*, 1989). Percentages of mitotic index (MI), indicative of mitotic activity, were calculated from mitotic cells in counted hepatocytes.

For the anticlastogenic assay, the protocol used in this study was modified from that described previously (Charoensin *et al.*, 2010). Male rats were divided into 4 groups. The pre- and co-treatments with 100–1,000 mg/kg of *C. nervosum* extract concomitant with 30 mg/kg diethylnitrosamine administration were performed on days 15 and 18. Partial hepatectomy was performed on day 22 and all rats were sacrificed by diethyl ether anesthesia and liver perfusion on day 26.

## Results

### Chemical constituents in an aqueous extract of *C. nervosum*

The total phenolic compounds were 181.16±0.59 mg GAE/100 g fw. The amounts of total flavonoids and condensed tannins were 54.86±3.45 mg CE/100 g fw and 1,902.72±183.63 mg CE/100 g fresh weight, respectively.

**Figure 1:**
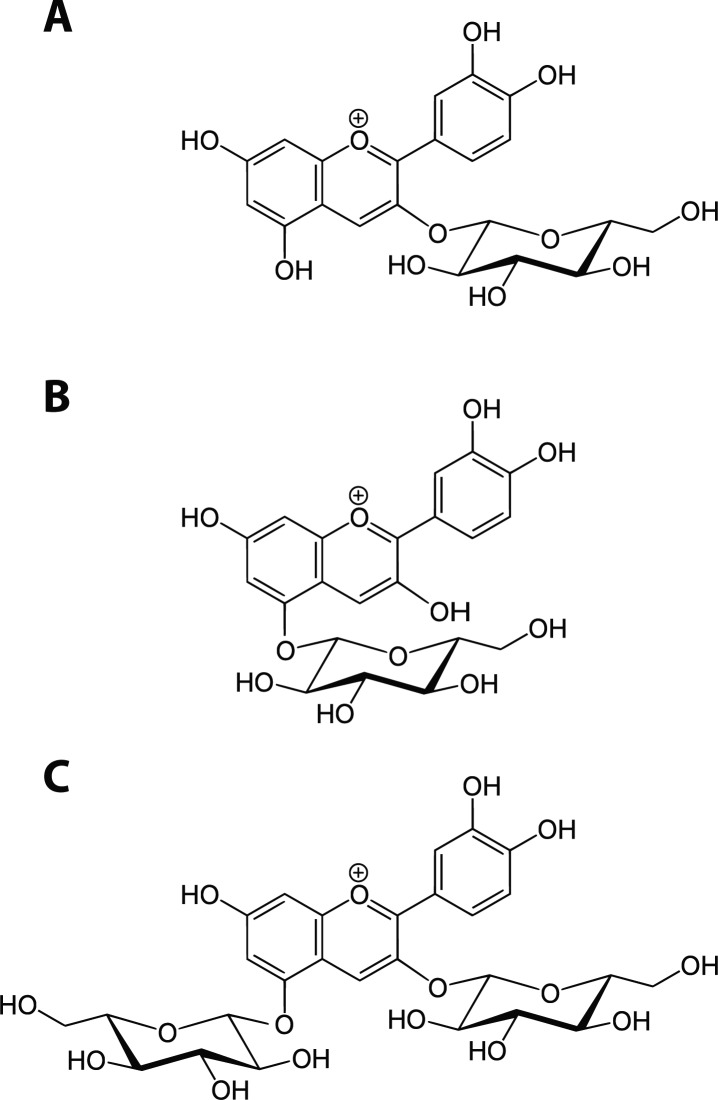
Chemical structures of cyanidin-3-glucoside (A), cyanidin-5-glucoside (B) and cyanidin-3,5-diglucoside (C).

The UV-Vis spectrum measured at 520 nm of *C. nervosum* showed three separate peaks at retention times 17.80, 20.83, and 42.82 min, which corresponded to anthocyanin constituents. Their LC-ESI-MS spectra indicated the structures consistent with the authentic standards which included cyanidin-3,5-diglucoside, cyanidin-3-glucoside, and cyanidin-5-glucoside, respectively (Figure 1). The percentage of relative contents among the assigned anthocyanins indicated that cyanidin-3-glucoside accounted for the major anthocyanin of *C. nervosum* with a relative content of 73.48±19.26% (Table 1).

**Table 1:** Anthocyanins identified in *C. nervosum* extract and their relative contents by LC-ESI-MS techniques.

Retention time(min)	Structural assignment of anthocyanins	[M]^+^	ESI-Mass spectrum	Relative Content in extract (%)
17.80	cyanidin-3,5-diglucoside	611	287, 449, 611	0.75±0.26
20.83	cyanidin-3-glucoside	449	287, 449	73.48±19.26
42.82	cyanidin-5-glucoside	449	287,449	25.77±19.01

### *In vitro* mutagenic and antimutagenic activities of aqueous extract of *C. nervosum*

For the test doses no killing effect or sign of cytoxicity was observed for either TA98 or TA100 strains of *S. typhimurium*. The numbers of TA98 and TA100 His^+^ revertant colonies from plates treated with 0.8–200 mg/ml of the extract under present and absent metabolic activation were not significantly different from negative control (Table 2). This suggested that the dose of 0.8–200 mg/ml of *C. nervosum* extract was non-mutagenic to the strains of *S. typhimurium,* either with or without metabolic activation. Considering the antimutagenicity of *C. nervosum* extract in the TA98 strain (Table 3), co-treatment of MeIQ and 100–200 mg/ml of the extract in the presence of S9 mix showed dose-dependent antimutagenic activity, and the highest dose exhibited significant reduction (*p*<0.05) of the percentage of revertant colonies (% inhibition = 62.3±18.0) in comparison with the positive control. However, the percentage of revertant colonies observed from 100–200 mg/ml of the extract co-treated with AF-2 in the absence of S9 mix was not significantly different from the positive control. The number of revertant colonies by co-incubation of AFB1 with the extract was slightly lower compared with the positive control, indicating a possible weak antimutagenic effect.

**Table 2:** Mutagenic activity for aqueous extract of *C. nervosum* based on non-metabolic (–S9) and metabolic activation (+S9) using *S. typhimurium* TA 98 and TA100 strain.

Treatment	Average of His^+^ revertant colonies			
	TA 98		TA100	
	– S9	+ S9	– S9	+ S9
AF-2 2 ug/ml	286±32.6	N.A.	834±184.3	N.A.
2-AA 10 ug/ml	N.A.	408±35.9	N.A.	612±186.9
DW 50 ul	32±6.3	37±9.4	143±34.8	180±36.7
*C. nervosum* extract 0.8 mg/ml	32±6.1	26±3.7	138±39.2	134±36.1
*C. nervosum* extract 4 mg/ml	32±9.0	24±3.7	137±41.7	135±38.0
*C. nervosum* extract 20 mg/ml	32±8.8	22±4.8	133±31.4	139±36.6
*C. nervosum* extract 100 mg/ml	31±8.0	24±1.7	141±44.2	129±32.2
*C. nervosum* extract 200 mg/ml	37±15	30±9.1	N.A.	N.A.

The His^+^ revertant colonies are expressed as Mean±SEM of three independent replicates.

AF-2; 2-(2-furyl)-3-(5-nitro-2-furyl)-acrylamide, 2-AA; 2-aminoanthracene, DW; distilled water, N.A.; not analyzed

**Table 3:** Antimutagenic activity assay for aqueous extract of *C. nervosum* against various mutagens using *S. typhimurium.*

Test compounds	Average of His^+^ revertant colonies		
	AF-2 1 ug/ml	AFB1 0.16 ug/ml	MeIQ 0.05 ug/ml
Standard mutagen	185±19.2	428±75.3	181±12.9
*C. nervosum* extract 100 mg/ml	178±17.5 (3.8)	314±43.7 (29.2)	134±6.0 (27.3)
*C. nervosum* extract 150 mg/ml	N.A.	254±41.6* (34.1)	69±3.5* (62.3)
*C. nervosum* extract 200 mg/ml	142±14.0 (23.8)	N.A.	N.A.

AF-2; 2-(2-furyl)-3-(5-nitro-2-furyl)-acrylamide, AFB1; aflatoxin B1, MeIQ; 2-amino-3,4-dimethylimidazo[4,5-f]quinoline, DW; distilled water, N.A.; not analyzed

The number of spontaneous revertant colonies range from 17±2.4–21±3.7. Parentheses indicated the percentage inhibition of the extract against each mutagen.

* Significant difference in comparison with positive control (*p<*0.05)

### Evaluation of acute toxicity of aqueous extract of *C. nervosum*

None of the animals showed any changes in general appearance after administration of 5,000mg/kg of the aqueous extract. There were no deaths during the 14-day observation period, and gross examination of internal organs of all rats did not reveal any abnormality.

### *In vivo* clastogenic and anticlastogenic effects of aqueous extract of *C. nervosum*

The clastogenicity of *C. nervosum* extract was examined by a micronucleus assay in regenerating rat livers. Four days after partial hepatectomy, the growth rate of the treated rats was not significantly different from that of the control group. The mean number of micronucleated hepatocytes and mitotic indices was not significantly different in rats treated with 1,000 mg/kg of the test compound compared with control rats (Table 4). This suggested that the *C. nervosum* extract was non-mutagenic in rat liver. While the biological activities of anthocyanins from fruits and vegetables have been extensively studied, their anticlastogenicity against diethylnitrosamine (DEN), a hepatocarcinogen in animals and humans, remains unknown. In the present study, the DEN-administered rats had significantly greater numbers of micronuclei and a higher mitotic index compared with control rats. The dosed rats had an average body weight similar to that of the control rats. The analysis of micronucleated hepatocytes of rats administered 100–1,000 mg/kg of *C. nervosum* extract showed that the numbers of micronucleated hepatocytes of DEN-treated rats were not affected by any of the doses of the extract tested compared with control rats (Table 4).

**Table 4:** Clastogenicity and anticlastogenicity of an aqueous extract of *C. nervosum* in livers of male wistar rats.

Treatment	MNHEPs/1,000	Mitotic index (%)
5% Tween 80	2.15±1.26	0.15±0.00
*C. nervosus* 1,000 mg/kg	2.68±0.84	0.29±0.26
DEN + 5% Tween 80	12.61±2.05*	0.50±0.27
DEN + *C. nervosum* 100 mg/kg	10.17±3.23	0.45±0.06
DEN + *C. nervosum* 200 mg/kg	10.15±2.05	0.38±0.17
DEN + *C. nervosum* 1,000 mg/kg	10.88±2.79	0.54±0.41

MNHEPs/1,000 (Micronucleated hepatocytes per 1,000 hepatocytes) are expressed as Mean±SD.

* Significant difference from negative control (5% Tween 80 as vehicle), *p*<0.05

## Discussion

Recently, our research group reported the effect of an aqueous extract of *C. nervosum* on antioxidant systems in the rat liver, including total glutathione, glutathione peroxidase, catalase, and heme oxygenase-1 activities, indicating a possible biphasic effect on oxidative status of the rat liver (Taya *et al.*, 2009). Subsequently, we studied the composition and *in vivo* biological effects of *C. nervosum* fruits related to their antioxidant properties. We found that the aqueous extract of *C. nervosum* was rich in cyanidin-3-glucoside with a relative content of 73.48±19.26%. The LC-ESI-MS spectra indicated that the structures were consistent with the authentic standards and those presented in previous reports, which included cyanidin-3,5-diglucoside, cyanidin-3-glucoside, and cyanidin-5-glucoside, respectively (Figure 1) (Tian *et al.*, 2005; Jansom *et al.*, 2008).

In the studies of *in vitro* mutagenicity and antimutagenicity, *C. nervosum* extracts were not only non-mutagenic but also antimutagenic. Co-treatment of MeIQ and the extract in the presence of S9 mix showed dose-dependent antimutagenic activity, and the highest dose exhibited significant reduction (*p<*0.05) of the percentage of revertant colonies in comparison with the positive control. The number of revertant colonies by co-incubation of AFB1 with the extract was slightly different compared with the positive control, indicating a weak antimutagenic effect (Table 3). Anthocyanins and phenolic compounds were reported to exhibit antioxidant, antimutagenic and anticarcinogenic properties (Caillet *et al.*, 2012; Galvano *et al.*, 2004). Our results agree with previous reports in which natural anthocyanins acted as antimutagens. “It has been reported that anthocyanins from colored maize modulate the mutagenic activity of 2-aminoanthracene (2-AA) in *S. typhimurium* TA98 and TA100 through the inhibition of base-changed mutation (Mendoza-Díaz *et al.*, 2012). Additionally, the anthocyanin colors from vegetables inhibited the reverse mutation induced by food mutagen 2-amino-1-methyl-6-phenylimidazo[4,5-b]pyridine (PhIP) in the presence of rat liver microsomal activation system in a dose dependent manner (Aoki *et al.*, 2004).”

With regard to these findings, the protective activity could be explained as the result of the major anthocyanins. The mechanism of inhibition of the mutagen-activating enzymes is the protection of the hot-spot region of the gene from mutation. Biotransformation of MeIQ and AFB1 are mediated by CYP1A2 and CYP3A4 in human and rat liver cells, respectively (Forrester *et al.*, 1990; Turesky *et al.*, 2002). Therefore, cyanidin glucosides in *C. nervosum* possibly modulate the key enzymes by means of activity inhibition.

While the biological activities of anthocyanins from fruits and vegetables have been extensively reported, their anticlastogenicity against diethylnitrosamine (DEN), a known hepatocarcinogen in animals and humans, has not previously been investigated. We found that DEN-administered rats had significantly greater numbers of micronuclei and higher mitotic indices compared with control rats (Table 4). This result was consistent with experiments using DEN as a clastogen inducing hepatic micronuclei in rats and mice (Tates *et al.*, 1980; Cliet *et al.*, 1989). DEN was thus used as a model inducer in the experiment for anticlastogenic activity of *C. nervosum* extract in rat liver. The analysis of micronucleated hepatocytes of rats administered *C. nervosum* extract showed that the extract did not affect the number of micronucleated hepatocytes of DEN-treated rats compared with control rats (Table 4). When consumed, anthocyanins are absorbed in the stomach of rats and appear in both portal and systemic plasma (Passamonti *et al.*, 2003). The anthocyanin concentration of systemic blood may reflect its distribution and existence in target organs, especially the liver as a major site for metabolism. Passamonti *et al.* proposed a portal blood-liver transport mechanism of anthocyanins, which was related with an organic anion carrier called bilitranslocase in HepG2 cells (Passamonti *et al.*, 2005). They also determined the concentration of anthocyanins in portal and systemic plasma and in liver of rats given a mixture of grape anthocyanins, suggesting that most anthocyanins were absorbed in parent structures. Due to the fact that the *in vitro* antimutagenic assay used was carried out by bacterial mutation, the biotransformation was not entirely similar to that occurring in test animals. Additionally, the influences of microbial metabolism after upper intestinal absorption and metabolizing enzymes in the liver, along with loss and decrease of bioavailability of the active compounds, were possible reasons for the differences in the results from *in vitro* and *in vivo* antimutagenic observations.

In conclusion, our results indicated that anthocyanins isolated from the *C. nervosum* var. *paniala* fruit were not only safe in acute toxicity test, but even displayed antimutagenicity *in vitro*. These observations will be beneficial for the development of this species as a medicinal plant.
